# Enhancing neutralizing antibodies against receptor binding domain of SARS-CoV-2 by a safe natural adjuvant system

**DOI:** 10.1016/j.virusres.2023.199047

**Published:** 2023-01-21

**Authors:** Maliheh Darvish, Zahra Moosavi-Nejad, Seyed Omid Ranaei Siadat, Fataneh Fatemi, Ali Khatibi

**Affiliations:** a Department of Biotechnology, Faculty of Biological Sciences, Alzahra University, Tehran, Iran; b Protein Research Center, Shahid Beheshti University, Tehran, Iran

**Keywords:** The receptor binding domain, Sodium alginate, Natural adjuvant, vaccine delivery, Surrogate virus neutralization test

## Abstract

The receptor binding domain (RBD) plays a pivotal role in the viral entry as it enables the engagement of severe acute respiratory syndrome 2 (SARS-CoV-2) with the human angiotensin-converting enzyme 2 (ACE2) receptor for host cell entry. RBD is the major target for developing viral inhibitors and vaccines. Expression of recombinant RBD in *E.coli* is highly scalable with a low-cost procedure despite its high expression level compared to expression in mammalian and yeast cells. Using an alternative natural adjuvant system instead of alum adjuvant, increased immunogenicity of RBD antigen in serological assay including direct ELISA and surrogate Virus Neutralization Test (sVNT) was demonstrated with high levels of IgGs and neutralizing antibodies in mice sera immunized with RBD:AlSa (Alum and Sodium alginate) formulation. The sVNT is a simple and fast test that can be used instead of the conventional virus neutralization test requiring live virus and BSL3 laboratory to detect total neutralizing antibodies against RBD. Additionally, results showed a safety profile for sodium alginate which supported using it as an alternative natural adjuvant.

## Highlights

• It is introduced a novel natural adjuvant for RBD-based vaccine for the first time.
• Sodium alginate will increase the adsorption efficacy of alum to RBD Antigen.
• Sodium alginate will increase the stability of recombinant vaccines, leading to enhancement of neutralizing antibody level.

## 1 Introduction

Coronavirus disease 2019 (COVID-19) is a novel potentially deadly infectious disease that was first reported in Wuhan, China which was confirmed to be caused by beta-coronavirus severe acute respiratory syndrome coronavirus 2 (SARS-CoV-2) (Wang et al., 2020). This virus has spread globally giving rise to the current pandemic. Since the beginning of the pandemic, there have been several variants of SARS-CoV-2. Vaccination against SARS-CoV-2 may prevent a generation of new variants and reduce the rate of disease outbreaks (Hassani et al., 2022). Although some mutations have been reported in the RBD sequence, it shows the major functionally neutralizing epitopes that make RBD be more immunogen, then cross-nAb (neutralizing antibody) titers will be increased with subsequent protection against viral variants (Consortium, 2020, Marnik, 2022, Zhang et al., 2020, Mallory et al., 2019, Schoeman and Fielding, 2019, Kleanthous et al., 2021). As well, some studies indicated that antibodies from the plasma of convalescents and the serum of vaccine-immunized individuals had neutralizing activity against RBD as a target (Hassani et al., 2022, Kleanthous et al., 2021, Fatemi et al., 2021).

SARS-CoV-2 genome is a single-stranded positive-sense RNA composed of 29903 nucleotides and 4 major open reading frames (ORFs) that encode structural proteins: the spike (S), the membrane (M), the nucleocapsid (N) and the envelope (E) proteins. Among these proteins, the S protein is essential for infection as it binds to the glycosylated ACE2 receptor. The S glycoprotein is composed of two subunits called the S1 and S2. RBD is related to residues 319-591 (223 aa) in the N-terminal of the S1 subunit and is responsible for the interaction with ACE2 on host cells. The RBD has a high affinity for ACE2 (Leandro et al., 2021, Fitzgerald et al., 2021, Kim et al., 2020, Walls et al., 2020). Antibodies against RBD can hamper the binding of RBD to ACE2 and entry of the virus followed by inactivating the viral cells by the humoral and cellular immune response. The SARS-CoV-2 RBD as an antigen for vaccine development, not only can induce neutralizing antibodies but can also be recognized by developed antibodies in the serum of convalescents (Shang et al., 2020, Li and Li, 2005).

To develop an efficient vaccine, it is necessary to assess some parameters such as using carriers and more potent immunogenic adjuvants as well boosters. Alum is a traditional adjuvant and one of the most common adjuvants used in vaccines. However, it has some caveats including difficult manufacturing, causing inflammation at the injection site, and resulting in precipitation and gross aggregation (Bello, 2009, Kato et al., 2010, Montomoli et al., 2011). To enhance immunogenicity and reduce the side effects of vaccines, researchers are interested in immune-modulating compounds from natural origin (Vasiliev, 2015, Raghuvanshi et al., 2001). Sodium alginate is the sodium salt of alginic acid, elicited from the cell walls of algae. Its ideal characteristics, nontoxic, nonirritant and biodegradable materials, make it a candidate of natural adjuvant for vaccine development (Montomoli et al., 2011, Borges et al., 2008, Abdelallah et al., 2016, Dobakhti et al., 2009, Li et al., 2008).

Previous studies have reported glycosylation sites in RBD which shows that there are no glycosylation sites in the middle and C-terminal regions of RBD. Also, it has been reported that neutralizing antibodies recognize this region to prevent the virus attack so we evaluated this region of RBD and expressed it in *E.coli* (Sui et al., 2004, Min and Sun, 2021). Using a different formulation of RBD antigen by the combination of aluminum hydroxide gel and sodium alginate and assayed it on mice by competitive ELISA and surrogate Virus Neutralization Test (sVNT), we were able to detect high titers of IgG in immunized mice sera as well as sera from COVID-19 patients. We tried to provide novel and useful insights for the production and formulation of RBD antigen as a vaccine candidate.

## 2 Materials and methods

### 2.1 Materials

The RBD gene was ordered to BIOMATIK. Plasmid extraction kit and gel recovery kit were purchased from GenAll. Enzymes and buffers for cloning were prepared from Thermofishers. The NTA-Ni resin was purchased from Sigma (USA). Mouse anti-His monoclonal antibody (mAb) HRP-conjugated, HRP-labeled goat anti-mouse IgG and HRP-labeled goat anti-human IgG were obtained from CMG (IRAN). Sodium alginate powder was from Sigma-Aldrich (USA). PT-SARS-CoV-2-Neutralizing-Ab-96 kit was provided by PISHTAZTEB Diagnostics (IRAN). Balb-C mice were purchased from Razi Vaccine & Serum Research Institute (IRAN). Animals have housed in accordance with WHO guidelines and standard laboratory conditions (WHO guidelines on nonclinical evaluation of vaccines 2005). Animal research were approved by Iran National Committee by Ethics in Biomedical Research.

### 2.2 Expression, on-column refolding and purification of recombinant RBD

The expression vector, Pet28a including the genetic sequence of 6X His-tagged RBD, was transformed into *E.coli* BL21 host bacteria. A single colony of the recombinant *E.coli* BL21 was inoculated into 5 ml LB containing 50 µg/ml kanamycin. The cell was incubated at 37°C overnight while shaking at 250 rpm. After cell growth, it was transferred to a 500 ml Erlenmeyer flask containing 100 ml of LB including 50 µg/ml kanamycin, followed by incubating in a shaker incubator at 37°C, 250 rpm (Fitzgerald et al., 2021). When the cell density of the culture reached 0.6-0.8 (OD_600_), the starting inoculum was transferred to a 2 L fermenter containing complex media: 42.85 g trypton, 21.4 g yeast extract, 47.33 g Na_2_HPO_4_, 45.33 g KH_2_PO_4_, 35.73 g NH_4_Cl, 9.44 g Na_2_SO_4_, 25 ml glycerol, 10 g glucose, 10 g lactose, succinic acid 500 mM, 0.03 g MgSO_4_ and trace elements based on Table 1 (Blommel and Becker, 2009, Briand et al., 2016).

**Table 1:** Stocks of trace elements for fermentation.

**Stock solution**	**Stock concentration**	**Material**	**ddH_2_O**	**Required volume**
FeCl_3_.6H_2_O	2.7%	0.06g	2ml	250 µl
CaCl_2_. 2H_2_O	15.8%	0.316g	2ml	250 µl
MnCl_2_.4 H_2_O	19.8%	0.396g	2ml	250 µl
ZnSO_4_.7H_2_O	28.8%	0.576	2ml	250 µl
CoCl_2_. 6H_2_O	4.76%	0.095g	2ml	250 µl
CuCl_2_. 2H_2_O	1.7%	0.034g	2ml	250 µl
NiCl_2_. 6H_2_O	4.76%	0.095g	2ml	250 µl
Na_2_MoO_4_. 2H_2_O	2.42%	0.048g	2ml	250 µl
H_3_Bo_3_	0.63%	0.012g	2ml	250 µl
Na_2_SO_4_	1.73%	0.034g	2ml	250 µl

Cell growth and auto-induction were done in a fermenter with a 2 L working volume, under the following conditions: 1 vvm airflow rate, the temperature of 37°C, pH 7.2, dissolved oxygen (DO) 30. After 24 h fermentation and protein expression, cells were harvested by centrifugation at 7000 rpm for 15 min at 4°C and stored at −20°C. A gram of cell pellet was suspended in 20 ml buffer A (Table 2), followed by sonication (7s pulse, 3s pause, 200 W for 20 times and 400W for 40 times). The lysed cells were centrifuged at 9000 rpm for 20 min at 4 °C and the inclusion bodies in the pellet were solubilized in 20 ml buffer B (Table 2) by sonication on ice (7s pulse, 3s pause, 400 W for 20 times) and incubated for 30 min at 4°C. The resulting lysate was clarified by centrifugation at 9000 rpm for 30 min at 4 °C, The supernatant was then filtered through a 0.22 µm membrane before being passed through a 10 ml Ni-NTA column. As described in method (Zhao et al., 2005), on-column refolding was done by step gradient concentration of buffer E including 4 M, 3 M, 2 M, 1 M, and 0 M urea (Table 2). The column was washed with 30 ml buffer F (Table 2) and eluted with 30 ml buffer G (Table 2). Collected fractions were verified using a 12% SDS-PAGE gel, purified fractions with bands corresponding to 28 kDa (expected size of the recombinant RBD) were pooled, adjusted to proposed concentration and dialyzed with normal saline(31,32).

**Table 2:** Buffers used for the refolding purification procedure and their volume required.

**Buffers name**	**Composition**	**Required volume**
Buffer A	50mM Tris-base, 500mM NaCl, 0.5% Triton X-100, pH 8	20 ml
Buffer B	100mM Tris-base, 4M Urea, pH 11	20 ml
Buffer C (equilibrating buffer)	50mM Tris-base, 500mM NaCl, pH 11	5 CV
Buffer D (binding buffer)	50mM Tris-base, 500mM NaCl,10 mM imidazole, 4M Urea, pH 11	4 CV
Buffer E (step gradient buffer including 4M, 3M, 2M, 1M, and 0M urea)	50 mM Tris-base, 500 mM NaCl, 5 mM imidazole, 20% glycerol, pH 11	2 CV of each molarity of urea
Buffer F (washing buffer)	50 mM Tris-base, 500 mM NaCl, 100 mM imidazole, 20% glycerol, 5% glucose, pH 11	3 CV
Buffer G (elution buffer)	50 mM Tris-base, 500 mM NaCl, 800 mM imidazole, 20% glycerol, 5% glucose, pH 11	3 CV

### 2.3 Western blot assay

The purified recombinant RBD proteins were separated by 12% SDS-PAGE and transferred onto the PVDF membrane. After blocking by PBS buffer (pH 8.0) containing 5% BSA for 1 h at room temperature, the membrane was incubated with anti His-tag HRP conjugated antibodies (1:2000 dilution) for 1 h at room temperature. Furthermore, the recombinant RBD on the PVDF membrane was detected by an anti-RBD antibody (1:1000 dilution) following anti-human IgG HRP conjugate (1:10000 dilution). After washing the membrane with TPBS buffer for 5 min, 3,3′-diaminobenzidine tetrahydrochloride (DAB) was applied for visualization (Mahmood and Yang, 2012).

### 2.4 UV absorption spectroscopy

The extinction coefficient (ɛ) of the folded protein is related to its amino acid composition. This value at 280 nm was predicted using the following equation:

ε=(nW×5500)+(nY×1490)+(nC×125)

Where *n* is the number of each residue, W is tryptophan, Y is tyrosine and C is cysteine in the RBD sequence. Using the estimated ɛ, the concentration of recombinant RBD was determined by UV spectrophotometry at 280 nm (BAS and KUIPERS, 2007).

Absorption spectra of native RBD and serum-antigen (native RBD) immune complex were obtained in the wavelength of 190-450 nm on Shimadzu UV-1700 spectrophotometer. Briefly, to form the immune complex, RBD antigen, with the concentration of 20 µg/ml, was incubated with mice sera isolated from each group of immunization, for two hours at 37°C followed by overnight incubation at 4°C (Raghav et al., 2017).

### 2.5 Vaccine formulation preparation and immunization

Sodium alginate (Sa) and Aluminum hydroxide gel (alum) were applied at concentrations of 5 mg/ml and 0.5 mg/ml, respectively which were prepared according to Abdel Allah et al. (2016) (Abdelallah et al., 2016). As shown in Table 3, different adjuvanted vaccine formulations were applied to reach a final concentration of 100 µg/ml RBD antigen. Alum-antigen and Sa-antigen mixtures were shaken overnight at 4 °C to ensure complete absorption. The formulation of alum and sodium alginate (AlSa) was prepared by adding alum to the antigen and shaking for two hours, followed by the addition of sodium alginate and shaken overnight at 4°C.

**Table 3:** Composition of adjuvant system formulations.

	1 (Negative Control)	2 (only antigen)	3 (Al)	4 (Sa)	5 (AlSa)
Normal saline	×				
RBD Ag (100 µg/ml)		×	×	×	×
Alum (0.5 mg/mL)			×		×
Sodium alginate (5 mg/mL)				×	×

Balb/c mice aged 4-6 weeks were randomly divided into five groups of 6 female mice as shown in Table 3. Five groups were vaccinated subcutaneously with 10 µg Ag/100 µl/mouse): 1. Normal saline as a negative control, 2. RBD Ag without adjuvant, 3.RBD Ag 100 µg/ml loaded on alum (Al), 4. RBD Ag 100 µg/ml in sodium alginate (Sa) and 5. RBD Ag 100 µg/ml loaded on alum and sodium alginate (AlSa) and boosted after 21 days with 10µg Ag/100µl/mouse. Blood samples were collected 10 days post-each immunization for detection of IgG (Borges et al., 2008, Song et al., 2022).

### 2.6 Evaluation of the loading efficacy in vaccine formulation

To assess the loading efficacy of each vaccine formulation, an indirect method was utilized in which the free antigen (RBD) remained in solution and was quantified by the Lowery method as described previously (Waterborg, 2009). For this purpose, an aliquot of each vaccine formulation namely, Al, Sa and AlSa, was centrifuged at 10000 rpm for 10 min and the concentration of antigen in the supernatant was determined using the BSA standard curve prepared simultaneously and the loading efficacy (LE) was calculated according to the following equation(22,23):

LE(%)=(totalamountofRBDAg−freeRBDAg)/(totalamountofRBDAg)*100.

### 2.7 Antibody titer by direct binding ELISA

The sera from blood samples were collected by centrifugation for 10 min at 14000 g. To measure the RBD-specific IgG in the serum sample, purified RBD (1µg/ml) was resuspended in 0.05 M carbonate buffer (pH 9.6) and dispensed into an ELISA plate 100 µl/well. After incubation of coated plate at 4°C overnight, it was then incubated with blocking buffer (PBS including 2%BSA) for 2 h at 37°C. The plate was washed by PBST (PBS+ 0.05% Tween20) four times and then 200 µl of the sera of each group (mice immunized with formulated antigen and the convalescent serum as positive control) was added to the first well of each column in 96-well plate and two-fold serial dilutions of each sample were prepared. Following incubation of the plate for 90 min at 37 °C, HRP-conjugated goat anti-mouse IgG antibody was added and incubated one more hour at 37 °C. TMB (3,3′,5,5′– Tetramethylbenzidine) was added (100 µl/well) as substrate after four washes with PBST. 50 µl of 0.2 M sulfuric acid was used to stop the reaction and the absorbance was read after 10 min at 450/630 nm using an ELISA reader (Biotech, USA) (Knezevic et al., 2022). The mean absorbance of the negative control wells was calculated. The highest dilution of each sample showing an absorbance above Cutoff, the mean + (3 × standard deviation) of negative wells, was considered as the antibody titer of that sample (Kumar et al., 2003, Frey et al., 1998).

### 2.8 Inhibition ELISA

The immunogenic specificity of the serum antibody against RBD antigen and the amount of inhibition was determined by the competitive or inhibition ELISA method (Raghav et al., 2017). Briefly, different amounts of antigen (concentration ranging from 0-20 µg/ml) was incubated with mice sera after immunization as well as serum of recovered COVID-19 patient at 37°C for 2 hrs followed by overnight incubation at 4°C. The antigen was coated in plate wells in the concentration of 1 µg/ml, the immune complex formed and thus was added into the well instead of the mice sera. The rest of the steps were performed the same way as indirect binding ELISA. Also as a control, each sera group was incubated with assay buffer instead of antigen. The coated wells incubated with the "No Antigen Control" (NAC) showed the maximum OD. The inhibition percent of different wells containing samples was calculated against the NAC wells using the following equation:

$$Inhibition\%=\left(A_{NAC}-A_{inhibited}\right)/A_{NAC}\times 100$$

where A_inhibited_ and A_NAC_ are the absorbance of inhibited and No Antigen Control wells (Sharma et al., 2019).

### 2.9 SARS-CoV-2 Surrogate Virus Neutralization Test (sVNT) in sera of immunized mice

sVNT was assayed according to the PT-SARS-CoV-2-Neutralizing-Ab-96 kit (from PISHTAZTEB Diagnostics). An ELISA plate from a kit was pre-coated with RBD antigen, 50 µl of positive control, negative control and the mice sera were added to each well with 2-fold dilution in duplicate. After 30 min of incubation at 37 °C, 50 µl of HRP-conjugated hACE2 was added to the wells, and mixed on shaker for 5 min. After 1 h of incubation and 4 times of washing with PBST, 100 µl of TMB as substrate was added. A colorimetric signal was developed after adding stop solution and read at 450 and 630nm. Inhibition percent was calculated by: 1-(sample absorbance value/negative control absorbance value) × 100 % Inhibition was plotted against reciprocal of dilutions for each sample. The reciprocal of dilution which results in a 50% inhibition, was described as the neutralizing antibody titer (IC_50_) (Tan et al., 2020, Sancilio et al., 2021, Hofmann et al., 2022).

### 2.10 In vivo safety assay

Immunized Mice in each group of formulation were monitored for 14 days and abnormal toxicity was assessed in each mouse. The clinical signs, local inflammation symptoms such as local swelling, redness and loss of hair at the site of injection, loss of body weight and death were monitored in each group for 14 days after first injection and boosters (Folegatti et al., 2020, Green and Al-humadi, 2013). The systematic reactogenicity was evaluated based on severity of edema and erythema in sites of injection and took score of 0 to 4 (Table 4) (Amokrane et al., 2017).

**Table 4:** Dermal observations.

Score	Grade	Edema	Erythema
0	None	No swelling	Normal color
1	Minimal	Slight swelling: indistinct border	Light pink: indistinct
2	Mild	Defined swelling: distinct border	Bright pink/pale red: distinct
3	Moderate	Defined swelling: raised border (˂1 mm)	Bright red: distinct
4	Severe	Defined swelling: raised border (≥1 mm)	Dark red: pronounced

### 2.11 Evaluation of cytokine levels in mice sera

The collected sera from each group of mice were assayed for measurement of anti-mouse cytokines levels such as INF-Ɣ, IL-4 and IL-10 using Invitrogen ELISA kit as instructions of the manufacturer.

### 2.12 Statistical analysis

All Data were expressed as the mean ± SD and analyzed using GraphPad Prism 9.0.0. One-way ANOVA and Two-way ANOVA Dunnett's multiple comparisons were used to compare the statistical significance of differences between cytokine and antibody levels of groups. Data were considered significant if p-values were <0.05.

## 3 Result

**Fig. 1:**
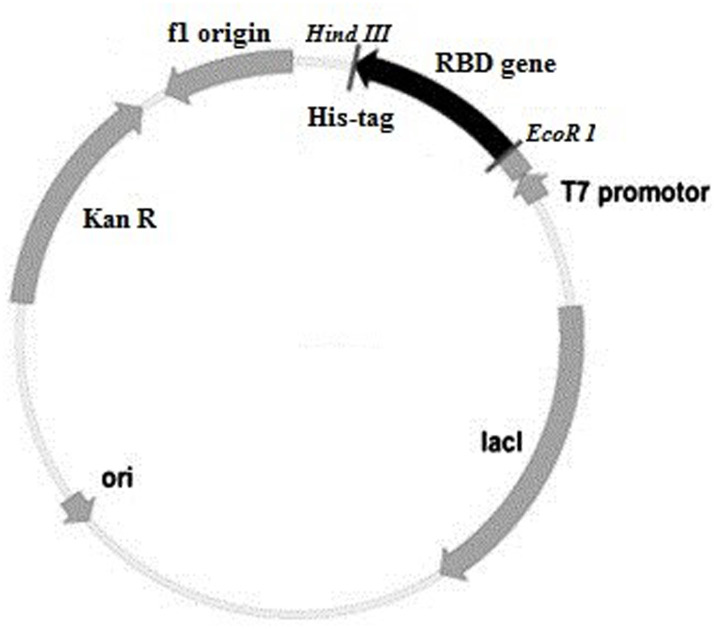
Recombinant plasmid pET28a-RBD, resistant to kanamycin, restriction enzyme EcoR I & Hind III.

**Fig. 2:**
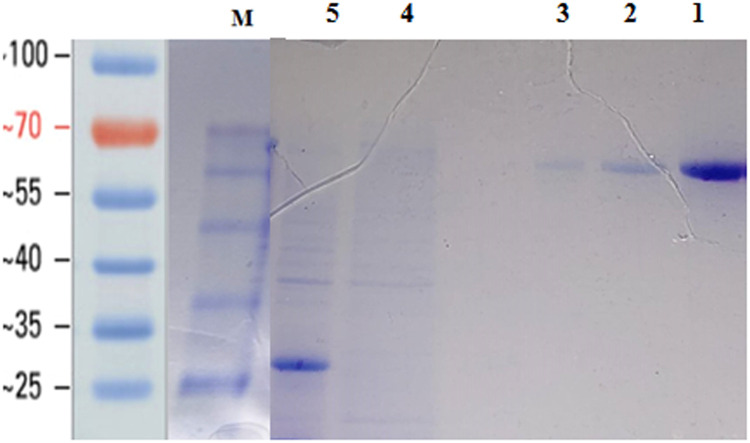
SDS-PAGE analysis of recombinant RBD of SARS-CoV-2 expressed in *Ecoli* BL21. The total proteins were extracted from *E.coli* cells. Lane (M): protein ladder (Thermo Sceintific), Lane (Wang et al., 2020): std BSA 10µg, Lane: std BSA 5µg, Lane (Hassani et al., 2022): std BSA 2µg, Lane (Consortium, 2020): negative control(*Ecoli* BL21 without RBD protein), Lane (Marnik, 2022): soluble recombinant RBD from cells extraction (2µl).

As shown in Fig. 1, the codon-optimized receptor binding domain of SARS-CoV-2 was cloned into pET28a, a vector for His-tag fusion protein expression. The recombinant RBD was overexpressed in *E.coli* BL21 cells as inclusion bodies by overnight auto-induction method. Integrity of expressed RBD was verified by their movement in SDS-PAGE based on molecular weight and further by immunological reactions with anti His-tag antibody on western blot (Fig. 2).

**Fig. 3:**
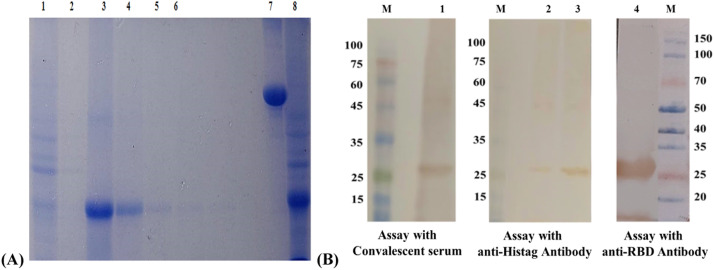
(A) Purification of RBD protein by NTA-Ni column, (Lane 1) Binding fraction, (Lane 2) Washing fraction, (Lane 3, 4, 5,6) elution Fractions including purified RBD, (Lane 7) BSA 10µg, (Lane 8) the cell lysate before loading on column (5µl). (B) Western blot assay of RBD protein. Purified RBD by NTA-Ni column was run in SDS-PAGE 12% with prestained protein marker (SMOBIO PM2500) in Lane (M), the gel was transferred on PVDF membrane and probed by convalescent serum from the COVID-19 patient (Lane 1), anti-His mAb (Lane 2 &3) and anti-RBD mAb (Lane 4). The amount of RBD loaded was 3µg in Lane 2, 5µg in Lane 1&3 and 15 µg in Lane 4.

After fermentation, cells were harvested and re-suspended in buffer A (Table 2), following several cycles of sonication and solubilization of inclusion bodies in lysis buffer (buffer B, Table 2). The lysate was clarified by centrifugation and evaluated by SDS-PAGE. According to Fig. 2, it is estimated that approximate 50% of total bacterial proteins are the recombinant RBD with predicted size of 28 kDa. The obtained total bacterial proteins were passed through a Ni-NTA column. As illustrated in Fig. 3A, most of the RBD proteins were bound to the column. Based on previous investigation (Yin et al., 2003, Mingcai Li, 2007), on-column refolding was done by passing a step gradient of urea (4,3,2,1 and 0 M) in refolding buffer (buffer E, Table 2). After washing unbounded contaminant protein from column with Buffer F, the RBD protein was eluted using Buffer G containing 800 mM imidazole (Table 2) (Fig. 3A). Expression yield and purification of recombinant RBD are summarized in Table 5.

**Table 5:** Yield of recombinant RBD purification from *E.coli*.

Purification steps	Total proteins (mg/l)^a^	RBD Ag (mg/l)^b^	Recovery rate (%)^c^
Cell lysate	1600	800	100
Inclusion body	702	692	44
Eluate	608	600	37.5

a The concentration of total protein was measured by Lowery method using bovine serum albumin as the standard protein.

b The amount of recombinant RBD Ag was estimated by densitometry method using SDS-PAGE & ImageJ software.

c Recovery rate (%): (recombinant RBD Ag in each purification step)/(recombinant RBD Ag in cell lysate) × 100.

### 3.1 Western blot

Identity of the recombinant RBD was performed by anti His-tag mAb, anti-RBD specific antibody and convalescent serum from one of the Covid-19 patients in Masih Daneshvari Hospital (Fig. 3B).

### 3.2 Adsorption of RBD Ag on adjuvants

**Fig. 4:**
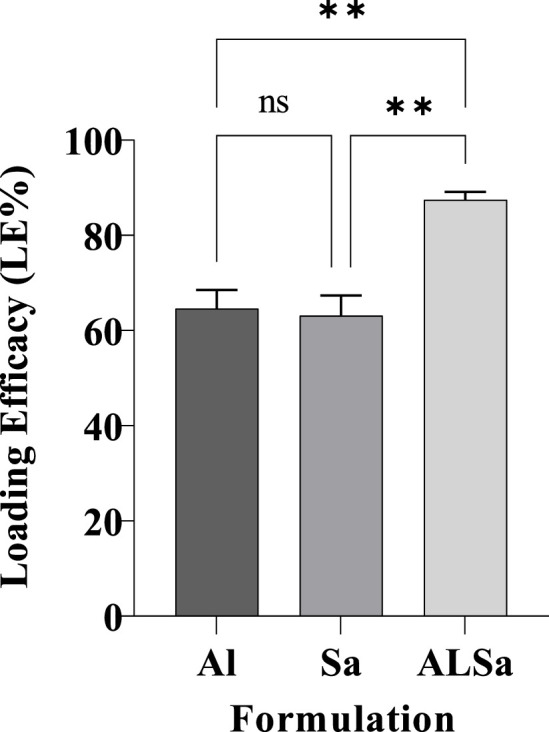
The Loading Efficacy (LE%) of RBD Ag in vaccine formulations. Double formulation containing alum & sodium alginate showed higher LE% compared to the single formulation containing alum or sodium alginate. ns: not significant, ** p< 0.01.

Evaluating the loading efficacy (LE) of RBD antigen on alum suspension formulations demonstrated that RBD antigen was efficiently adsorbed to alginate particles according to measured loading efficacy. The LE% of Al, Sa and AlSa was measured at 64.6 %, 63 % and 87.5%, respectively (Fig. 4). Therefore, it is confirmed that the addition of sodium alginate to alum did not diminish the adsorption of antigen to the alum. In contrast, a new adjuvant containing alum and sodium alginate together showed a significantly positive effect on adsorbing of RBD antigen (p<0.01).

### 3.3 Spectroscopic analysis

**Fig. 5:**
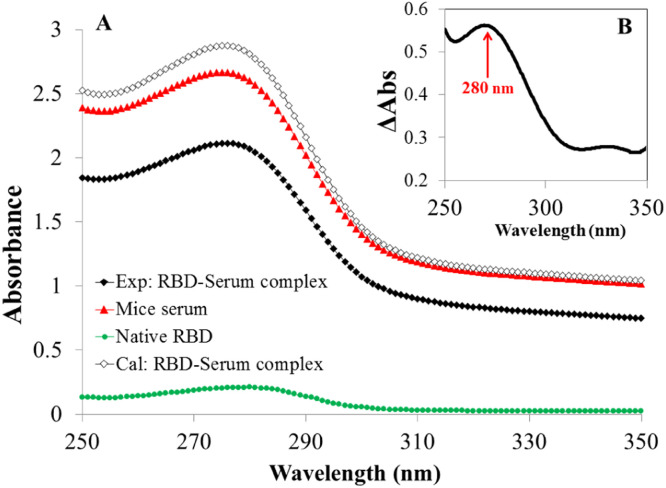
(A) UV absorbance spectrum of the mice serum alone, native RBD (20µg/ml) and RBD-serum immune complex experimentally (shown as Exp) and computationally (shown as Cal). (B) Difference between the serum absorbance and the immune complex absorbance on 250-350 nm shows increased absorbance of the serum sample at 280nm compared to the immune complex experimentally.

The UV-absorption profile of recombinant RBD, Serum from immunized mice with RBD:AlSa formulation and antigen-serum immune complex were recorded in a wavelength range of 250-350 nm as shown in Fig. 5. The recombinant RBD is included by a high content of tyrosine amino acids (2 Tryptophan, 15 Tyrosine, and 7 Cysteine), as it showed maximum absorbance at approximately 280 nm. The absence of a typical slope between 300 to 350 nm suggested that the protein did not form soluble aggregates (Fig. 5). According to the protein sequence, the extinction coefficient was estimated as 34,225 M^−1^cm^−1^. The absorbance of 1 mg/ml protein solution at 280 and 330 nm were 2.1 and 0.261, respectively, then by ɛ_280nm_ and corrected Abs_280nm_, the concentration of recombinant RBD was calculated as approximately 53.7 µM.

UV profile of the immune complex shows a decrease in absorbance as compared to spectra of immunized mice serum (Fig. 5). Serum from mice immunized with RBD antigen showed maximum absorption on the wavelength of 280nm due to the existence of all aromatic residues (Lys, Trp & Phe) involved in antibody epitopes (Fig. 5A). However, the existence of RBD antigen in immune complex (mixed with the serum sample) and binding to anti-RBD IgG in the serum, caused 3D structural changes of IgG and engagement of epitopic residues, then the position of aromatic amino acids was altered and the immune complex showed less absorbance on 280nm compared to the mice serum. Whereas if there wasn't the specific IgG against RBD antigen in the immunized mice serum and the interaction between antigen-antibody did not occur, the absorbance of the RBD-Serum complex would be increased computationally compared to the serum alone as shown in Fig. 5A.

### 3.4 Humoral immune response

**Fig. 6:**
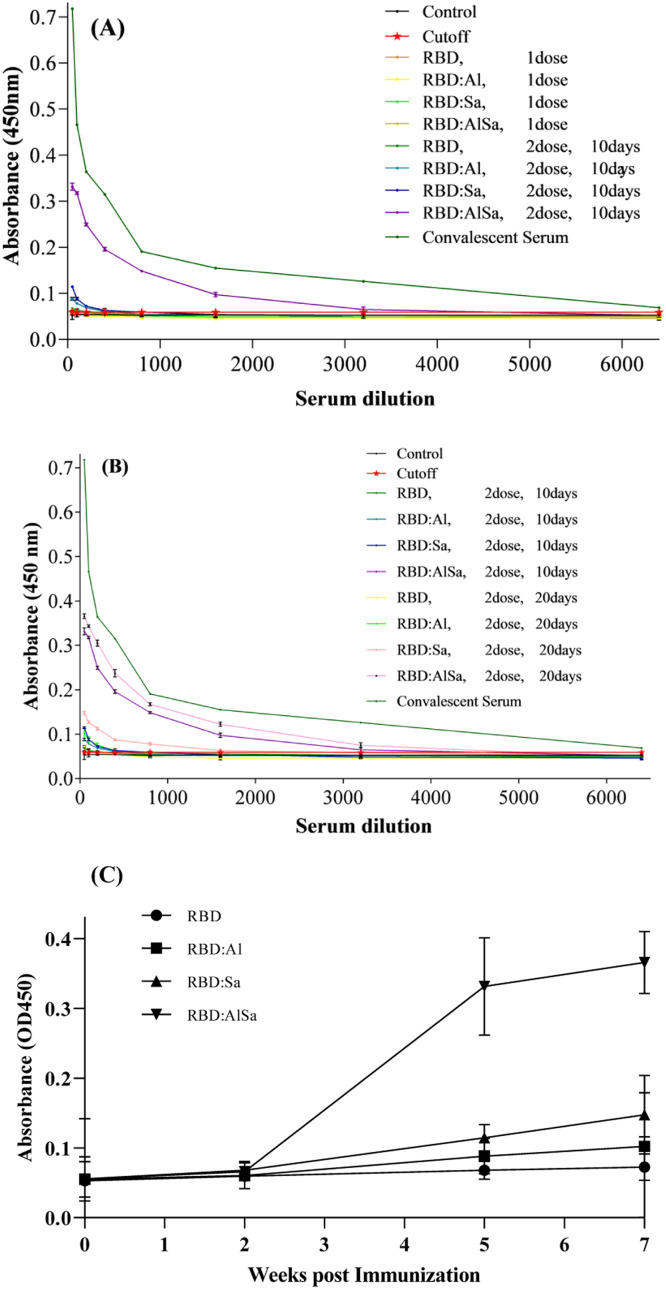
Anti-RBD IgG titers of mice immunized with different vaccine formulations. (A) Sera was collected 10 days post first and boost injections and serum anti-RBD total IgG was determined. (B) Serum titers were analazed for different formulations 10 & 20 days post socend injection. The convalescent serum was considered as a positive control in graphs A and B. (C) Time intervals of RBD-specific IgG production in mice immunized. Bleeding of immunized mice with three groups of the formulation was done at different time points and the trend of anti-RBD Ab production was assayed by ELISA.

In order to investigate humoral responses induced by the recombinant RBD antigen, mice sera from each group were collected 10 days after the first and second vaccination and 20 days after the booster shot and the anti-RBD IgG levels were analyzed. RBD antigen associated with sodium alginate combined with alum induced a strong immune response. As shown in Fig. 6A,B, the formulation of AlSa induced higher RBD-specific IgG levels than alum and alginate-adjuvanted formulations suggesting synergism between alum and sodium alginate. Furthermore, antibody titers of samples collected from mice vaccinated with RBD:AlSa formulation, 20 days post second immunization, were significantly higher than samples collected 10 days after the second immunization (Fig. 6A,B). So, booster dose injection and blood sampling at a longer time interval after the second dose injection showed that the antibody titer increased and was close to the antibody titer of the positive control (convalescent sera). As shown in Fig. 6C, the trend of anti-RBD IgG production in mice immunized with RBD:AlSa was increased more than twice in mice immunized with RBD:Sa and more than 3 times in mice immunized with RBD:Al and RBD after 7 weeks.

**Fig. 7:**
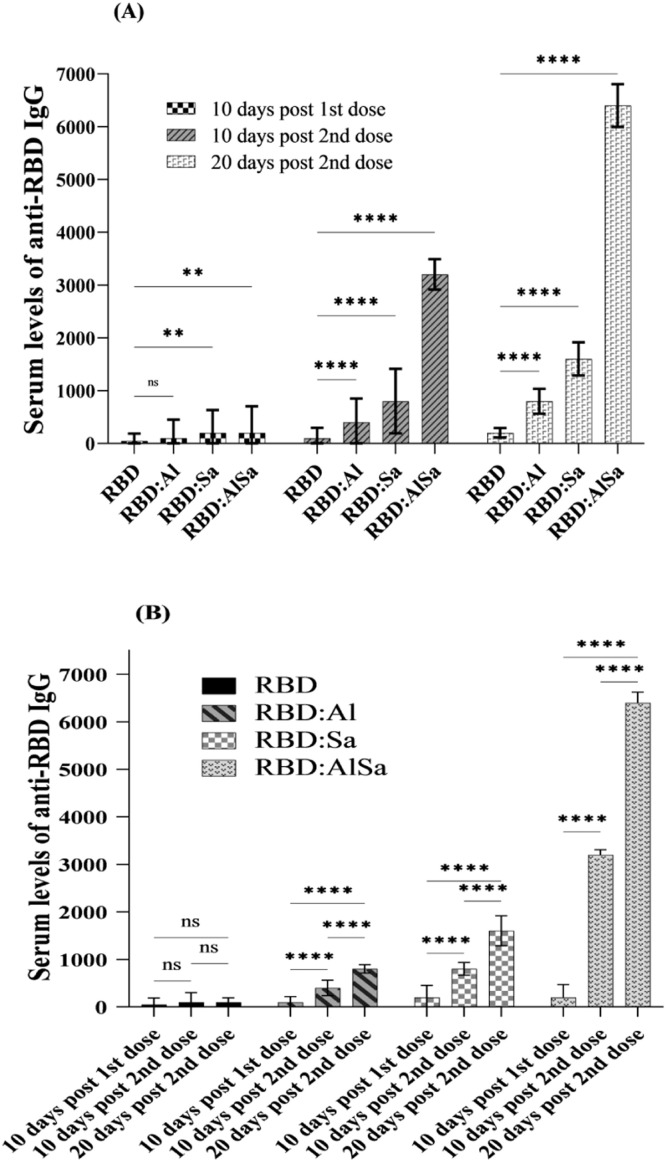
RBD Ag-specific total IgG levels of mice immunized with single and double adjuvanted-vaccine formulations. (A) The plot shows the difference in IgG levels of single and double formulations at different times of injection. (B) The plot shows the IgG level increasing of each Al, Sa & Al/Sa adjuvanted vaccine after 10 & 20 days post booster injection. ns refers to not significant and **** p<0.0001.

Furthermore, the level of IgG (the highest dilution above cut-off) against RBD Ag, 20 days post vaccination, in the serum of immunized mice with RBD:AlSa was shown a significant difference with p<0.001 compared to other formulations. However, the IgG level in mice serum immunized with RBD Ag without adjuvant showed no significant difference (ns) 10 and 20 days after booster vaccination (Fig. 7A,B).

**Fig. 8:**
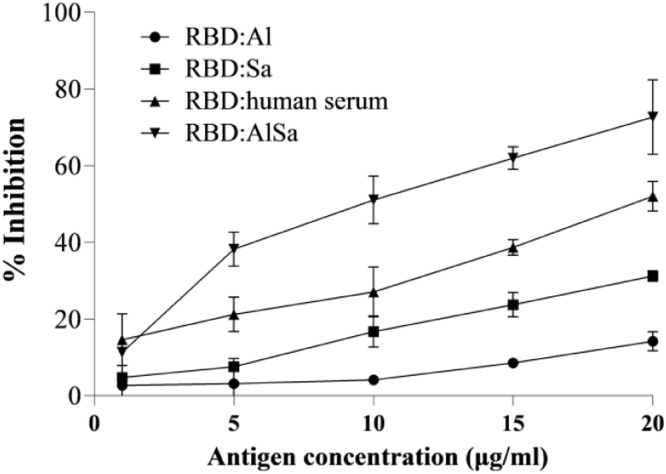
Competitive ELISA showing percent inhibition for the interaction of anti-RBD in immunized mice sera and recovered person's serum with RBD antigen.

Competitive ELISA is recommended as an alternative test for verification and specificity of induced antibody titer in the immunized mice sera. Immunized mice with RBD:AlSa formulation demonstrated inhibition of 72.6% with an RBD concentration of 20 µg/ml. However, two other formulations containing either alum or sodium alginate as adjuvant showed maximum inhibition of 14.16 % and 31.18 %, respectively, at the same RBD concentration (Fig. 8).

Forming the immune complex of the recombinant RBD and antibody included in the serum of the recovered person from COVID-19 showed about 52% of inhibition (Fig. 8) so it mentioned a close relation between the recombinant RBD and induced antibody against SARS-CoV-2.

### 3.5 sVNT

**Fig. 9:**
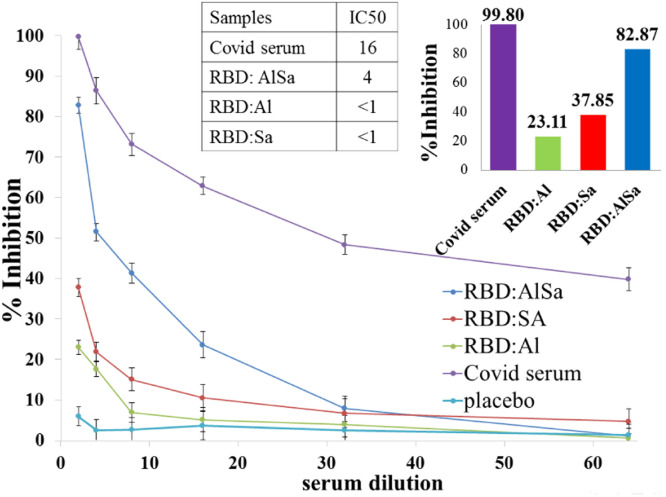
Neutralizing antibody titer of Immunized mice sera with RBD:Al, RBD:Sa, RBD:AlSa, control serum and serum from the COVID-19 case. Reduction of % inhibition with a dilution of sera (1:2, 1:4, 1:8, 1:16, 1:32, 1:64) is shown.

Analysis of the sera from mice immunized with three types of RBD formulation and the sera from convalescent showed that interaction between hACE2 and Spike protein of SARS-CoV-2 can be inhibited in a dose-dependent manner (Fig. 9). No evidence of inhibition was shown in a control group of mice. Analysis of the sample from convalescent indicated the expected pattern of results with the highest neutralizing antibody titer (IC_50_). In this study, IC_50_ is reported as a dilution that resulted 50% inhibition and this serum dilution is considered as the neutralizing antibody titer for each sample. Neutralizing antibody titers of the convalescent sample was 4 times more than sera from mice immunized with RBD:AlSa formulation as shown in the table in Fig. 9. However, sera samples from mice group immunized with RBD:Al and RBD:Sa showed % inhibition Less than IC_50_ at low dilutions. Although, the comparison of all samples (at dilution 1:2) was demonstrated that the inhibition percent of the neutralizing antibody in mice sera immunized with RBD:AlSa (83 %) was close to the inhibition percent of the convalescent serum (100%) (Fig. 9).

### 3.6 In vivo safety

Each group of mice immunized subcutaneously with three adjuvanted formulations was observed for 14 days. Neither mortality nor weight loss was observed in the immunized mice groups. Furthermore, no signs of hair loss and local swelling were seen at the site of injection with either formulation containing natural adjuvants or alum-adjuvanted formulations. According to Table 6, except for a limited number of mice, the rest did not demonstrate any edema or erythema effect on the injection sites.

**Table 6:** Summary of reactogenicity frequency*.

**Group of immunization**	**RBD**	**RBD:Al**	**RBD:Sa**	**RBD:AlSa**
**Animal per group**	6	6	6	6
**Edema severity**				
**0**	6	5	6	6
**1**	0	1	0	0
**2**	0	0	0	0
**3**	0	0	0	0
**4**	0	0	0	0
**Erythema severity**				
**0**	6	5	5	6
**1**	0	1	1	0
**2**	0	0	0	0
**3**	0	0	0	0
**4**	0	0	0	0

⁎ Number of mice showing the reactogenicity score during the study.

### 3.7 Cytokine levels

**Fig. 10:**
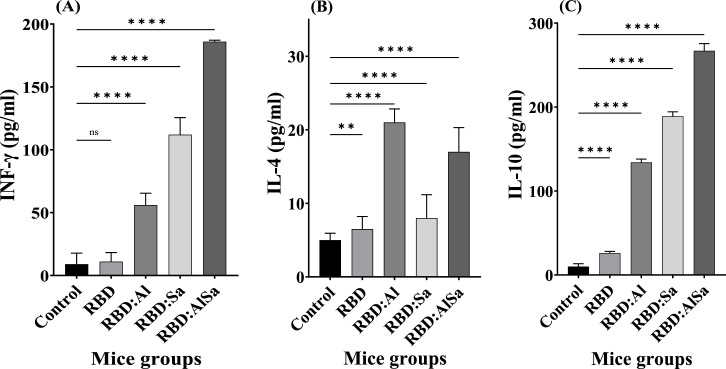
levels of INF-γ (A), IL-4 (B) and IL-10 (C) in mice sera vaccinated with different formulations of RBD Ag. were determined by ELISA method. *ns* refers to not significant, ** p<0.01 and **** p<0.0001.

The cytokine levels were measured in the mice sera by ELISA method. The highest level of IL-4 was related to the mice immunized with a single adjuvant of Alum that showed the value of 21 pg/ml significantly different from the control and other groups (p<0.0001). However, mice vaccinated with double adjuvant system, AlSa, demonstrated the highest levels of IL-10 and INF-γ, respectively with the value of 267 pg/ml and 186 pg/ml compared to control, unadjuvanted RBD and single adjuvanted RBD groups with significant difference (p<0.0001). Additionally, production of INF-γ and IL-10 were increased significantly in the mice sera immunized with single adjuvanted RBD with alum and sodium alginate relative to the control and unadjuvanted RBD group (p<0.0001) (Fig. 10).

## 4 Discussion

In this study, we expressed the RBD of SARS-CoV-2 efficiently in a prokaryotic system with high yield by auto-induction methodology. The estimated molecular weight of the recombinant His-tagged RBD was confirmed as 27 kDa by SDS-PAGE and western blot.

High expression of RBD protein in bacterial cells resulted in RBD inclusion body. To solubilize RBD proteins in high concentration, we used urea (4 M) in lysis buffer and to eliminate urea and obtain purified RBD protein with native conformation, purification and refolding of soluble RBD proteins was done simultaneously on-column with gradient removing of urea. The native protein stability was maintained by adding cosolvents such as glycerol and glucose to refolding and elution buffers.

RBD of the S protein is regarded as a significant target of the humoral immune system and has the ability to induce potent neutralizing antibody responses. To enhance immunogenicity of RBD protein, a new adjuvant was developed. The safety and the immunogenicity of the new adjuvant system, sodium alginate, as well as combining it with alum, were compared with adjuvant alum. Sodium alginate, a natural source of vaccine adjuvant, was proven as a safe pharmaceutical ingredient by FDA. Some researchers used alginate nanoparticles as a novel adjuvant and vaccine delivery system. Sarei and *et al*. increased immunogenicity tenfold by designing an alginate delivery system for diphtheria toxoid. As well, in some studies, researchers have utilized sodium alginate as a carrier and encapsulation agent to protect protein-based antigens from digestive enzymes (Sarei et al., 2013, Xu et al., 2022). In this study, RBD proteins in three types of formulation systems including either alum or sodium alginate in the single formulation as well as double formulation of alum and sodium alginate were assessed in vivo in terms of safety and immunogenic response. The results showed that double formulation showed significantly higher levels of IgG compared with the single formulation of alum or sodium alginate (Fig. 7). Sodium alginate adjuvanted RBD vaccine was demonstrated to be safe in double or single forms of the formulation. Moreover, the combination of alum and sodium alginate as adjuvant protects the antigen from degradation in vivo and enhances the potency of the vaccine. HBV vaccine (Fendrix®) and HPV vaccine (Cervarix®) are examples of this kind of adjuvant system that are already licensed in Europe. In addition, in a previous study, the potency and immunogenicity of the Hepatitis B vaccine were enhanced by using sodium alginate and chitosan as a safe natural adjuvant system (Abdelallah et al., 2016).

An important parameter in vaccine formulation is the adsorption efficacy of the alum-adjuvanted vaccine. According to the world health organization (WHO) guidelines for vaccine development including pharmacopeia, the adsorption of antigen to aluminum adjuvants have to be evaluated prior to administration due to the loading efficacy is effective for increasing the immunogenicity and essential in protecting the vaccine from the degradation of antigen after administration. In this study, the addition of sodium alginate to alum-adjuvanted vaccine resulted in efficient loading of RBD antigen in the formulation (Fig. 4). As predicted, double formulation of alum and sodium alginate demonstrated the highest degree of adsorption and loading efficacy, therefore it is regarded as the most immunogenic formulation.

In our study, three groups of mice were immunized subcutaneously with doses of 10 µg RBD Ag conjugated with either alum or sodium alginate and both. Among the examined mice, a double formulation of alum and sodium alginate induced higher levels of IgG titers compared to the single formulations. Additionally, booster injection of double-formulated vaccine enhanced titers of anti-RBD Ag about 16 folds higher than the first injection dose. Humoral immune responses to COVID-19 vaccines or natural infection were investigated by neutralization antibody titration as a significant indicator of protective immunity. To assay the specificity of induced antibodies in the immunized mice sera, sVNT was done. As shown in other studies, the RBD direct ELISA is not able to differentiate between total binding antibodies and neutralizing antibodies. However, employing sVNT based on RBD is a more specific assay to reliably quantify RBD-based neutralizing antibodies. The sVNT assay of RBD formulated with AlSa showed more than 80% inhibition and verifies results from competitive ELISA in which interaction between induced antibodies and RBD antigen demonstrated percent inhibition of about 80%, as well results mention neutralizing antibodies diagnose specifically RBD epitopes and will allow effective immune responses against virus cells. According to recent research have been done on immunization against SARS-CoV-2, sVNT can be as specific as cVNT (the conventional virus neutralization test), more sensitive, safer and rapidly conducted without using a live virus and biosafety level 3 (BSL3) compared to other methods to assay neutralization antibodies (Tan et al., 2020, Gazumyan et al., 2020).

The effect of sodium alginate formulated RBD vaccine on cellular immune response, and levels of cytokine produced by Th1 and Th2 responses were determined. The Th1 and Th2 responses are shown by the production of IFN-γ and IL-4 & 10, respectively. Our results mentioned an increase in the IFN-γ levels but a decrease significantly in the production of IL-4 in the double formulation of RBD with AlSa compared to alum-adjuvanted RBD. A significant increase in IL-4 value in mice sera immunized with single formulated with alum compared to single formulated with sodium alginate as well as an enhancement of IgG titer in mice vaccinated with sodium alginate adjuvanted RBD compared to alum-RBD, supported the use of sodium alginate alone as an alternative natural safe adjuvant instead of alum for RBD vaccine (Figs. 8 and 10). This finding was confirmed by previous publications (Abdelallah et al., 2016). Low levels of IL-4 and high levels of IFN-γ post vaccination with RBD-AlSa formulation suggest strong Th1 responses and the highest IgG2a/IgG1 ratio. The addition of sodium alginate to alum-adjuvanted RBD induced a qualitative shift in IgG titers and encouraged both humoral and cellular immune responses.

## 5 Conclusion

In summary, the recombinant RBD as a vaccine candidate against SARS-CoV-2 was produced by an effective and high yield auto-induction method in a prokaryotic system. Our study introduced a novel natural adjuvant for an RBD-based vaccine for the first time. Sodium alginate, as a safe and immunogenic adjuvant, in combination with aluminum hydroxide (double adjuvant) can induce the humeral and cellular response against the RBD antigen more increasingly compared to the conventional adjuvant, Alum. The RBD antigen in the double adjuvant formulation with strong adsorption demonstrated an expansive effect on the neutralization antibody production as it was approved by a surrogate Virus Neutralization Test (sVNT). This study will provide an opportunity for further research to improve the platform of the subunit-based vaccine. Although, more investigation is required for the long-term safety and clinical trials to make this type of vaccine formulation with sodium alginate applicable.

## CRediT authorship contribution statement

**Maliheh Darvish:** Conceptualization, Methodology, Software, Investigation, Writing – original draft, Visualization, Formal analysis. **Zahra Moosavi-Nejad:** Supervision, Project administration, Data curation, Validation, Formal analysis. **Seyed Omid Ranaei Siadat:** Conceptualization, Resources, Supervision, Project administration, Funding acquisition. **Fataneh Fatemi:** Validation, Writing – review & editing. **Ali Khatibi:** Software, Writing – review & editing.

## Declaration of Competing Interests

The authors declare that they have no known competing financial interests or personal relationships that could have appeared to influence the work reported in this paper.

## Data Availability

The authors are unable or have chosen not to specify which data has been used.
